# Influence of Sires on Population Substructure in Dülmen Wild Horses

**DOI:** 10.3390/ani14192904

**Published:** 2024-10-09

**Authors:** Silke Duderstadt, Ottmar Distl

**Affiliations:** Institute of Animal Breeding and Genetics, University of Veterinary Medicine Hannover (Foundation), 30559 Hannover, Germany; silke.duderstadt@tiho-hannover.de

**Keywords:** genetic diversity, paternal half-sib groups, birth cohorts, Merfelder Bruch, Dülmen, Croÿ, substructure, stallions, effective population size

## Abstract

**Simple Summary:**

Dülmen wild horses have been managed by the Dukes of Croÿ for about 180 years in the Merfelder Bruch, a region near Dülmen in Westphalia, Germany. The Dülmen wild horses are freely roaming in this area of about 1400 acres all year round without human intervention and have to cope with the harsh natural conditions. Each breeding season 2–3 stallions are employed to sire foals. Herdbook records for this herd are not available due to the wildlife living conditions, free from any human disturbances. In this present study, we analyzed the distribution of the male progeny by their sires and whether sire effects on the genetic diversity and structure in the male progeny can be found. The genetic substructure in female progeny could not be evaluated as the fillies could not be captured for sampling. The paternal half-sib groups showed a very similar genetic diversity, but pairwise genetic distances and neighbor-joining dendrograms indicated a clustering by sires and a marker variance of 9% between paternal progeny groups. Bayesian cluster analysis supported subdivision into paternal progeny groups. Cluster memberships to paternal progeny groups, which were sired in the same year but by different sires, may also be related to mares, while clusters of paternal progeny from the same sire, but in intervals of more than one year, could not be distinguished. The employment of sires which are able to withstand the harsh natural conditions may also ensure a high genetic diversity in their progeny.

**Abstract:**

The objectives of the present study were to analyze the influence of the stallions employed in the Dülmen wild horses on the genetic diversity and population substructure using Bayesian cluster analysis. The Dülmen wild horse is maintained as a unique horse population exposed to the natural conditions all year round in the Merfelder Bruch near Dülmen in Westphalia, Germany. Stallions selected for breeding have to prove their abilities to survive under this harsh environment. We used multilocus genotypic information from a set of 29 autosomal microsatellites to determine the paternity of 185 male foals sired by nine stallions. As females could not be sampled, we could not make inferences on all yearlings and test whether there are differences in the genetic population parameters between both sexes. The mean number of progeny was 19.92 with a range of 2–32, caused by the length of the service period per stallion. The average observed and unbiased expected heterozygosity was 0.688 and 0.631, the mean number of alleles was 4.448, and Wright’s F_IS_ was −0.173. Pairwise genetic distances (F_ST_ and Nei’s unbiased genetic distances) were significant and varied between 0.038 to 0.091 and 0.085 to 0.290, respectively. Neighbor-joining dendrogram plots clustered a large proportion of the paternal progeny groups in different branches. Posterior Bayesian analyses using seven paternal half-sib groups with 10–74 members supported a maximum of six clusters, with two paternal progeny groups not differing, and a median of five clusters, with two groups of two sires each falling into the same clusters. When sires were employed in non-consecutive years, progeny from these different years of the same sires were grouped in the same cluster, whereas the progeny of one sire from two consecutive years were in different clusters. We were able to distinguish male progeny from Dülmen wild horse stallions and to show the effects of stallion use on the genetic substructure in the Dülmen wild horse herd. In conclusion, the analyses showed the genetic potential of the Dülmen wild horse stallions to maintain a high genetic diversity and also the effects in which breeding seasons and for how long stallions are used to sire foals. The selection of stallions may be sensitive for the further development of genetic diversity and preserve this closed population as a valuable resource for further studies on the evolution of the horse.

## 1. Introduction

The Dülmen wild horse population is maintained as a freely roaming wild herd of horses in the wooden and marsh area of the Merfelder Bruch near Dülmen in Germany. The history of these horses dates back to the Middle Ages [1]. There were also other wild horse populations in Germany, but all of these other herds were given up in the early 19th century [1]. With the foundation of the Dülmen wild horse population as early as 1856, these horses are managed by the Dukes of Croÿ. A herdbook for this population has not yet been established, as matings, the birth of foals, and mares cannot be registered. The reason for this is that this herd should preserve its natural behavior and therefore no human interventions in this herd take place. The female population of Dülmen wild horses has been closed since 1856. Stallions used in this herd are selected from the Dülmen wild horse population; Mongolian, Exmoor, and Polish Konik stallions were also used in the past. Particularly, from 1957 to 2004, Polish Konik stallions were used alongside Dülmen wild horse stallions. Responsibility for this population and all breeding decisions have been in the hands of the Dukes of Croÿ for about 180 years. The primary aim of the Dukes of Croÿ is to preserve this wild horse population as a free-roaming herd, living all year round on pasture land with forest and without any human intervention for more than 180 years. In this way, the horses would retain their natural behavior and their ability to survive in the same natural conditions as in past centuries. The only human interference in this herd consists in the selection of stallions and the determination of the duration of their breeding period as well as in the yearly removal of all young males from the herd. The Dülmen wild horse population is not divided into individual groups, but the horses form family groups with 6–12 members and a lead mare. Females do not become pregnant before the age of four, and reproduce up to the age of thirty [1]. During the breeding season, only two stallions are used in the Dülmen wild horse herd at the same time for a limited period of 2–6 weeks. All selection decisions for the stallions are the responsibility of the Dukes of Croÿ. Each year, about 60–130 mares give birth to a foal. Young stallions that are selected as future potential stallions are reared together in a group, separately from the herd in the Merfelder Bruch, but under the same harsh environmental conditions. Stallions that do not show mating behavior or do not succeed in covering mares or are exhausted after a short time in the herd or lose condition are immediately removed from the Dülmen wild horse herd [1].

In the present study, we analyzed the influence of the sires on the genetic diversity of the Dülmen wild horse population and tested whether sires create a substructure in this population. We used 29 autosomal microsatellite markers (STRs) [2,3,4]. In addition, we included the genotyping data of Dülmen wild horses from our previous study on this wild horse population [4] and supplemented these previous data with the data of 93 additional horses. In previous studies, it was shown that this marker set is well suited to studying genetic diversity and admixture in horse populations [2,3] and also in Dülmen wild horses due to its high information content [4]. Microsatellite markers were also commonly employed to study genetic diversity and population substructure [5,6,7,8,9,10,11,12,13,14,15,16,17,18,19]. We should be able to investigate whether the allele frequencies and genetic distances between the stallions used in the Dülmen wild horse population are large enough to distinguish clusters of paternal progeny groups. A lack of subclustering may indicate that allelic diversity between stallions is low and even allele frequencies between stallions and mares are rather homogeneous. In addition, the natural selection of foals may contribute to subclustering, as inbreeding depression may be detrimental to foal survival if resilience and natural adaptation to cold and wet climates are reduced in inbred foals.

## 2. Materials and Methods

### 2.1. Ethical Approval

This study was approved by the Institutional Review Board of the University of Veterinary Medicine Hannover (Foundation) and the German Federal State office from North Rhine-Westphalia (registration number 8.84-02.05.20.12.066) on 17 April 2012.

### 2.2. Sample Collection

Hair root samples were collected from 185 male Dülmen wild horses at the yearly auctions. The samples of the Dülmen wild horses were taken from five different birth cohorts (2002 and 2011–2014) in the Merfelder Bruch. The samples from the birth year of 2002 were provided by a commercial lab (Certagen, Rheinbach, Germany) with the permission of the Duke of Croÿ. The foals were sired by 7 Dülmen wild horse stallions, which were selected from the herd in the Merfelder Bruch, and 2 Polish Konik stallions from Popielno, Poland. Per season, two (2011 and 2014) or three (2001, 2010, and 2012) stallions were breeding approximately 60–130 mares. Two stallions were used in two breeding seasons and one stallion in three consecutive breeding seasons. The distribution of the sampled horses by their year of siring, birth, and auction is given in Table 1. On average, 36% of foals born alive were sampled. Fillies are not captured at auctions or at other cases and are thus not accessible for research. In the 2013 birth year, fewer foals were born alive than expected. The reason for this lower number could be that 2/3 stallions had very few offspring in this birth year (Table 2). In the present study, we supplemented the 101 samples with 38 samples from the 2002 birth year, 13 samples from the 2011–2013 birth years, and 33 samples from the 2014 birth year. In addition, samples from all 9 stallions were collected. The sampling of mares and fillies was not possible as the female animals have to be kept away from human contact in accordance with the regulations for this herd [4].

### 2.3. DNA Extraction and Microsatellite Analysis

The preparation of genomic DNA from hair root samples using proteinase K digestion for the ethanol extraction and genotyping of 29 autosomal microsatellite markers was performed following sample collection. The 29 microsatellite markers used are identical to the marker set from our previous study [4]. Protocols for genotyping and scoring were also described in previous studies [2,3,4]. This marker set includes the markers AHT34, ASB17, COR007, COR017, COR018, COR022, COR024, COR045, COR056, COR058, COR069, COR070, COR071, COR082, HMS07, HTG03, HTG06, LEX07, LEX33, LEX34, LEX63, LEX68, SGCV16, SGCV28, TKY19, CA425 (UCDEQ425), UM011, VHL20, and VHL209. In brief, we used multiplex PCRs with fluorescently labelled primers and the PCR products, after dilution with formamide loading dye in ratios from 1:4 to 1:16, were used for gel electrophoresis on a LI-COR 4300 DNA analyzer (LI-COR Biosciences, Lincoln, NE, USA). Allele sizes were determined by the use of reference markers with known fragment lengths and 100 bp molecular weight standards (LI-COR Biosciences) on each gel [4].

### 2.4. Statistical Analysis

#### 2.4.1. Genetic Diversity and Population Differentiation

The identification of possible genotyping errors due to null alleles was performed using Micro-Checker software, version 2.2.0.3 (University of Hull, Kingston upon Hull, UK) [20]. We used the software Cervus, version 3.0.7 (Field Genetics, London, UK), to determine the paternity for each male foal [21]. The most likely sire among the possible candidates is determined by the highest logarithm of a likelihood ratio (Lod score). Deviations from Hardy–Weinberg Equilibrium (HWE) were determined across all loci in each population using Chi-square tests (SAS/Genetics, version 9.4, Statistical Analysis System Institute, Cary, NC, USA, 2024). In addition, we determined polymorphism information content (PIC) and allelic diversity (AD) for each marker over all populations. Allele frequencies, the number of alleles (N_A_), the mean number of alleles per locus (MNA), the number of effective alleles (N_e_), observed (H_o_) and expected (H_e_) heterozygosity, unbiased expected (uH_E_) heterozygosity, Shannons’s information index (I), the number of private alleles (PA), and Wright’s F_IS_, F_IT_, F_ST_, and Nm (Nm = ((1/F_ST_) − 1)/4), number of migrants) [22] were calculated using GENEALEX, version 6.502 [23]. Molecular genetic relationships among populations were derived using Wright’s F_ST_ [22], Nei’s standard genetic distance (D_S_), and Nei’s unbiased genetic distance (D_US_) [24]. The neighbor-joining tree topology was obtained with DARrwin, version 6.0.021 (CIRAD-BIOS UMR AGAP, Montpellier, France) [25], using Nei’s standard genetic distance. We used NE-ESTIMATOR version 2 (University of Queensland, Brisbane, Australia) [26], a tool for multi-locus genotypes, to produce estimates of contemporary effective population sizes. Effective population size (N_eff_) is an important parameter for the evaluation of endangered populations, particularly when only a part of the population is sampled [26,27]. We used the approach based on linkage disequilibrium, which is well suited for microsatellite data and provides precise estimates for populations with N_eff_ < 200 [26]. The effective population size is an important criterion for deriving the endangerment status of populations [28]. Populations with Neff < 50 are highly endangered and appropriate breeding management is necessary for their conservation. An effective population size of >50–100 should ensure that the detrimental effects of inbreeding cannot threaten the conservation of such populations [29]. In addition, we tested for recent bottlenecks using BOTTLENECK, version 1.2.02 (INRA, Montpellier, France) [30], under a stepwise mutation model.

#### 2.4.2. Assessment of Population Structure and Admixture

We employed posterior Bayesian clustering methods developed by Pritchard et al. [31] and Falush et al. [32,33] with the software STRUCTURE 2.3.4 (Stanford University, Stanford, CA, USA) to infer the genetic substructure of the Dülmen wild horse population and degree of admixture by paternal half-sib groups. We excluded 2 half-sib groups with 2 and 5 samples only because unequal sampling may influence posterior probabilities and may introduce bias in estimating the highest number of clusters [33,34,35,36,37,38]. In addition, we genotyped more than 20 markers to increase the power for finding the uppermost level of population structure [33,38]. The program STRUCTURE has implemented a Markov chain Monte Carlo (MCMC) approach to identify groups of individuals at Hardy–Weinberg and linkage equilibrium. The most likely membership to a group is calculated as the natural logarithm of the probability (P) of the observed genotypic array (G), given the preassigned number of clusters in the data set ln P(G|K). The most likely number of clusters corresponds to the maximum of the likelihood function ln P(G|K). We ran STRUCTURE in 20 independent repetitions for each K from 1 to 9 to obtain a representative set of values of the likelihood function ln P(G|K). The number of iterations in each run was 10^6^ after a burn-in length of 10^5^ iterations. We chose an admixture model with a correlated allele frequencies model and a uniform prior. Individual admixture alpha was set to be the same for all clusters. The ΔK method was applied to detect the uppermost hierarchical level of the genetic partitioning of the data [39]. Therefore, we calculated the mean ln P(K) and its standard deviation, ln′(K), |ln″(K)|, and ΔK statistics from 20 repeated runs with the software STRUCTURESELECTOR (Chinese Academy of Sciences, Shandong, China) [40]. The ΔK method was developed to improve the detection of clusters and this method performs well when equal sampling in subpopulations is ensured [38]. However, when sampling is uneven, the ΔK method does not recover the uppermost level of population structure. In this case of uneven samples for subpopulations, four alternative statistics (MedMean K, MedMed K, MaxMed K, and MaxMean K) were proposed by Puechmaille (2016) [38]. These methods use either the median (Med) or the maximum (Max) of the proportion of membership for each cluster (K = 1–9 or K = 1–12), calculated from the 20 replicates, and are less influenced by uneven sampling. The threshold for the membership coefficient above which a population was considered as a member of a cluster was set at 0.5. The maxima of these values for all pre-assigned clusters were chosen as an estimate of the number of clusters that can be distinguished in the present data set. A graphical representation of the results, including the population cluster stratifications with CLUMPAK [41], mean ln P(K), the standard deviation (sd) of ln P(K), ln′(K), |ln″(K)|, ΔK, MedMed K, MedMean K, MaxMed K, and MaxMean K, was done using STRUCTURESELECTOR [40].

## 3. Results

### 3.1. Characteristics and Genetic Diversity of the Microsatellite Markers

There were no markers with a significant deviation from HWE and no loci with null alleles. The least polymorphic markers were COR022, LEX034, and SGCV28 with four alleles (N_A_) per locus over all samples and the most polymorphic markers were COR056 and LEX033, each with eleven alleles per locus (Supplementary Materials Table S1). The mean N_A_ was 6621 ± 0.345 alleles. The sum of all alleles over all loci amounted to 192. Observed heterozygosity (H_o_) ranged from 0.2595 (HTG006) to 0.8865 (VHL020). Measures of population differentiation Wright’s F_ST_ ranged from 0.062 (HTG006) to 0.187 (VHL209). Measures of marker informativeness (PIC) and allelic diversity (AD) ranged from 0.2257 (HTG006) to 0.8154 (COR056) and from 0.2359 (HTG006) to 0.8350 (COR056), respectively. Average PIC and AD across all markers were 0.626 and 0.666, respectively. The mean F_IT_ coefficient was −0.022 across all markers and varied between markers from −0.181 to 0.137; the corresponding value for the F_ST_ coefficient was 0.126 and ranged from 0.062 to 0.222.

### 3.2. Pedigrees of Stallions and Distribution of Progeny by Stallions

**Figure 1:**
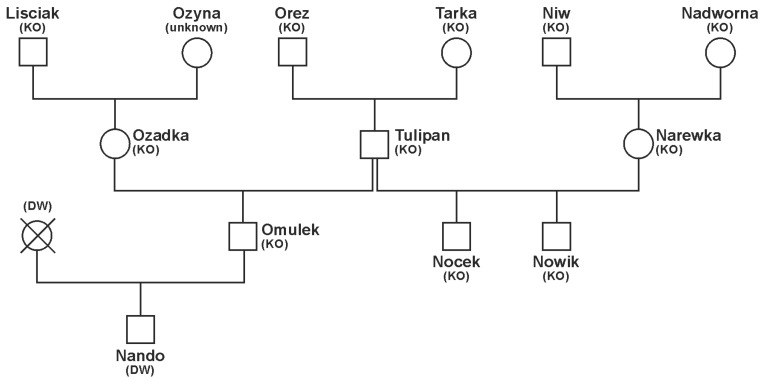
Pedigree of the stallions Nocek, Nowik, and Nando, who sired foals in the Dülmen wild horse herd in the Merfelder Bruch. The dams are Polish Konik mares (KO) or a mare with unknown breed (unknown) or a Dülmen wild horse mare (DW).

**Figure 2:**
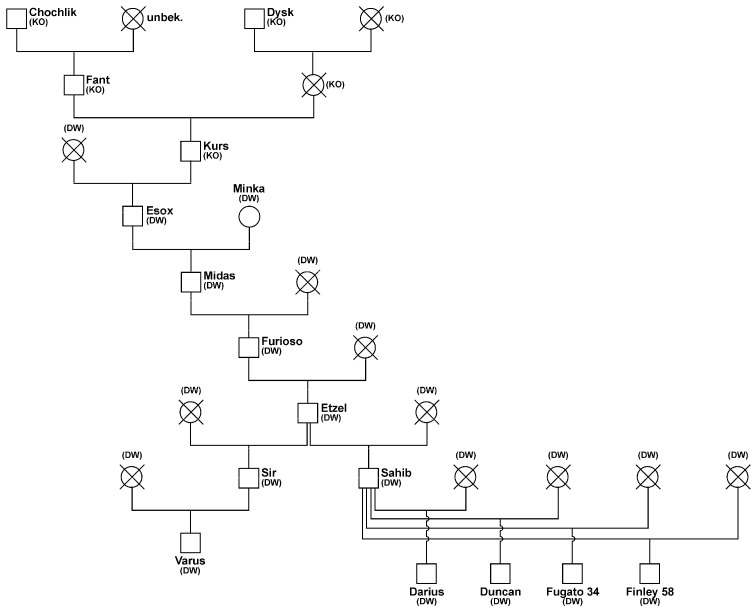
Pedigree of the stallions Sahib, Darius, Duncan, Fugato 34, Finley 58, and Varus, who sired foals in the Dülmen wild horse herd in the Merfelder Bruch. The dams are Polish Konik mares (KO) or an unknown mare (unbek.) or Dülmen wild horse mares (DW).

The pedigrees of the nine stallions employed in the breeding seasons 2001 and 2010–2013 are shown in Figure 1 and Figure 2. The stallions Nocek and Nowik are Polish Koniks and full-siblings by Tulipan and Narewka, whereas Nando is an F_1_-progeny of the Polish Konik sire Omulek and a Dülmen wild horse mare. 

The stallions Darius, Duncan, Fugato 34, and Finley 58 are paternal half-siblings by Sahib and grandsons of Etzel. These four half-sibs received an expected proportion of 1.5625% genes of the Polish Konik sire Kurs via their paternal path. The dams are unknown Dülmen wild horse mares (DW). Varus is also a grandson of Etzel and received an expected proportion of 1.5625% genes from the Polish Konik sire Kurs. The stallions Darius, Duncan, Fugato 34, Finley 58, Sahib, and Varus sired foals in the breeding seasons 2001 and 2010–2013.

The distribution of the progeny by stallions and birth years is shown in Table 2. The number of foals sired correlates with the period of use of stallions in the Dülmen wild horse herd in the Merfelder Bruch (Supplementary Materials Table S2) and with the ranking between the stallions at the same time in the herd. Nando, Darius, and Fugato 34 were used for 5 days (2010), 12 days (2012), and 11 days (2012) in the herd, whereas the duration of use for the other stallions and Fugato 34 in 2013 was, on average, 30.5 days (range: 15–40 days).

### 3.3. Genetic Diversity of Paternal Half-Sib Groups

Observed and unbiased expected heterozygosity in paternal offspring groups of Dülmen wild horses varied from 0.666 (Fugato 34) to 0.731 (Darius) and from 0.616 (Finley 58 and Fugato 34) to 0.686 (Darius) (Table 3). The mean number of alleles across paternal half sibs was 4.448 with a range of 2.310 (Nando) to 5.897 (Duncan), which corresponds to the number of progeny in this study. Private alleles (1–4) were found in six out of nine paternal half-sib groups. The mean F_IS_ coefficient was −0.173 and varied from −0.111 (Fugato 34) to −0.412 (Nando).

**Figure 3:**
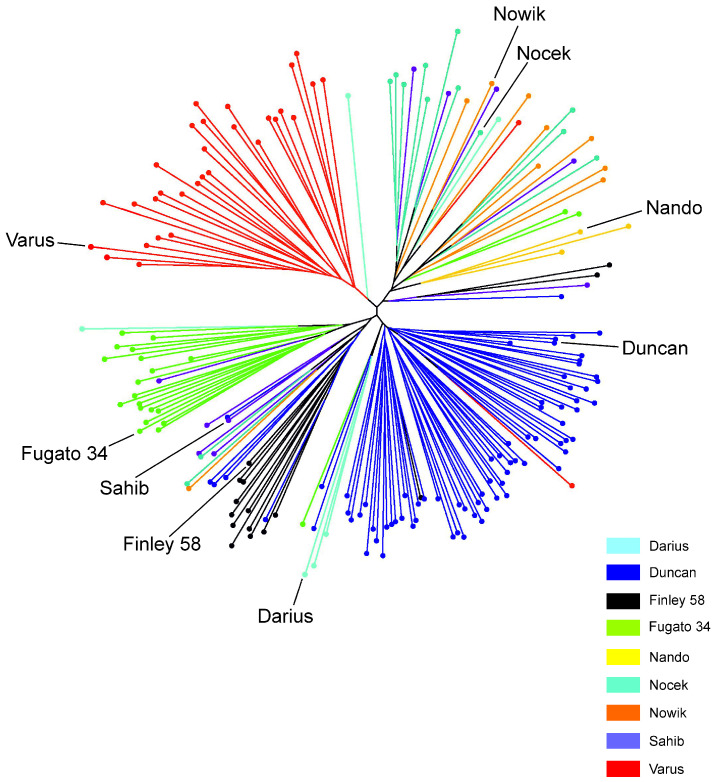
Individual-animal-based neighbor-joining dendrogram for the progeny groups of the nine stallions and the nine stallions themselves. Progeny groups and their sires are represented in the same color.

We tested progeny group means for significant differences (Supplementary Materials Table S3). Observed, expected, and unbiased expected heterozygosity were not significantly different between sire group means. Significant differences for the diversity measures influenced by the size of progeny groups were mainly caused by the progeny of Nando and Darius, because the smaller number of offspring resulted in lower values for MNA, N_e_, and I. Pairwise F_ST_ estimates among the nine paternal half-sub groups are shown in Supplementary Materials Table S4. The lowest differentiation was seen between the progeny of Sahib and Duncan (0.038), as well as between the progeny of Nocek and Nowik (0.04). Genetic differentiation with F_ST_ estimates were largest between the progeny of Nowik, Varus, and Nocek on the one hand and the progeny of the other sires on the other hand, when omitting the progeny of Nando. The high estimates of genetic distances with the progeny of Nando are caused by the low number of progeny of this sire. For Nei’s standard genetic distances (D_S_) (Supplementary Materials Table S5) and Nei’s unbiased genetic distances (D_US_) (Supplementary Materials Table S6), similar patterns for progeny groups with 10 and more progeny were obtained. The highest estimates for the Nei’s unbiased genetic distances were obtained between the progeny of Fugato 34 and Nowik (0.257), between the progeny of Varus and Fugato 34 (0.250), and between the progeny of Finley 58 and Varus (0.241). In addition, we calculated the relative variance proportions between the seven progeny groups with 10 and more offspring (Supplementary Materials Table S7). The largest proportions of the inter-stallion variances were between the Polish Konik stallions Nocek and Nowik on the one hand and all remaining five stallions (8–14%). The smallest estimate (4%) was obtained between the full-sibs Nocek and Nowik. The branches of the individual-animal-based neighbor-joining dendrogram for the half-sib groups split by paternal progeny groups with few exceptions (Figure 3).

### 3.4. Genetic Diversity of Birth Cohorts

Marker diversity by birth cohorts showed slight variation for the observed and unbiased expected heterozygosity in the range from 0.660 (2014) to 0.694 (2002) and from 0.627 (2012) to 0.660 (2002), respectively (Table 4). There were no significant differences in the measures for genetic diversity between means of birth cohorts, except for the F_IS_ estimates between 2012 and 2014 (Supplementary Materials Table S8). In addition, we tested for recent past bottlenecks using Wilcoxon tests under a stepwise mutation model (SMM) for heterozygosity excess, deficiency, and excess or deficiency with BOTTLENECK version 1.2.02 [30]. We could not detect significant test results (*p* values > 0.05) for the three Wilcoxon tests for an excess or deficiency of heterozygosity in the different birth cohorts.

Pairwise F_ST_ estimates among the five birth cohorts are shown in Supplementary Materials Table S9. The largest differentiation was seen among the progeny of the birth cohorts of 2012 and 2014 (0.041), 2013 and 2014 (0.036), and 2002 and 2012 (0.034). The lowest pairwise F_ST_ estimate was between the 2011 and 2013 birth cohorts. The same pattern for Nei’s standard genetic distances (Ds) (Supplementary Materials Table S10) and Nei’s unbiased genetic distances (D_US_) (Supplementary Materials Table S11) between birth cohorts was obtained. The estimates for Ds between birth cohorts of 2012 and 2014, 2013 and 2014, and 2002 and 2012 were 0.168, 0.145, and 0.135, respectively. The corresponding estimates for D_US_ were 0.144, 0.114, and 0.111, respectively. The lowest estimates for F_ST_, Ds, and D_US_ were seen between 2011 and 2013 birth cohorts with values of 0.017, 0.064, and 0.037, respectively. The branches of the individual-animal-based neighbor-joining dendrogram for the birth cohorts are mixed between birth years (Supplementary Materials Figure S1).

### 3.5. Population Structure and Admixture

**Figure 4:**
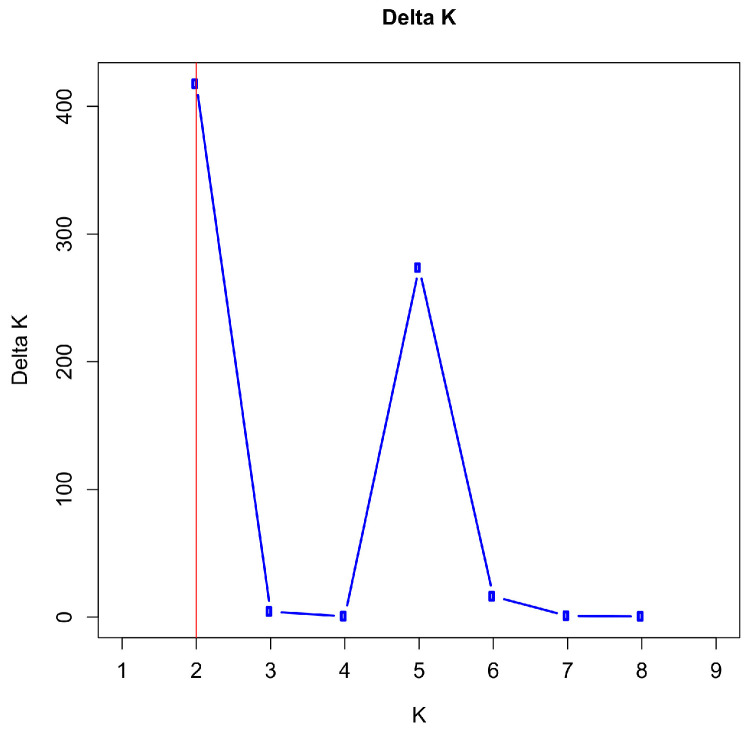
Plot of ΔK (Delta K) values from 20 independent STRUCTURE runs for K = 1–9 using 29 microsatellite markers of seven paternal half-sib groups of Dülmen wild horses. The maximum value was achieved by K = 2, which is indicated by a red vertical line, and the next highest value was achieved at K = 5.

The most likely number of clusters was two as determined by the maximum value for ΔK between K = 2 and 9 (Figure 4 and Supplementary Materials Table S12). With K = 2, ΔK reaches its maximum value with 417.56 and the next highest value at K = 5 with 273.59. The mean of the likelihood function reaches its maximum with −21316.85 at K = 5 (Supplementary Materials Figure S2). For runs with other K values, ΔK reached much smaller values.

**Figure 5:**
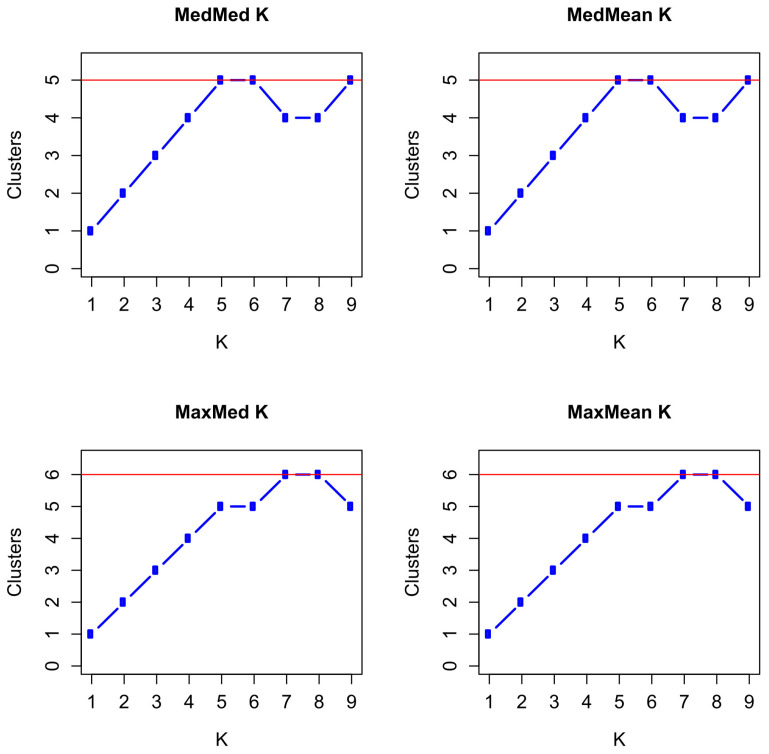
Plots of the medians (Med) and means (Mean) of the median (Med) and maximum (Max) number of inferred proportions of memberships to clusters of seven paternal half-sib groups of Dülmen wild horses with a threshold of 0.5. The red line indicates the maximum values reached by the MedMed K, MedMean K, MaxMed K, and MaxMean K statistics.

For the statistics MedMed K and MedMean K, we obtained maxima for K = 5, 6, and 9 with five clusters, and for the statistics MaxMed K and MaxMean K, we obtained maxima for K = 7 and 8 with six clusters at a threshold of 0.5 (Figure 5 and Supplementary Materials Table S13. Increasing the threshold to 0.6, resulted in MedMed K and MedMean K with K = 5 and 6 in five clusters, and in MaxMed K and MaxMean K with K = 7 in six and five clusters, respectively. With a threshold of 0.7, the maxima were obtained at K = 5 with five clusters for MedMed K and MaxMed K, but with four clusters for MedMean K and MaxMean K. 

**Figure 6:**
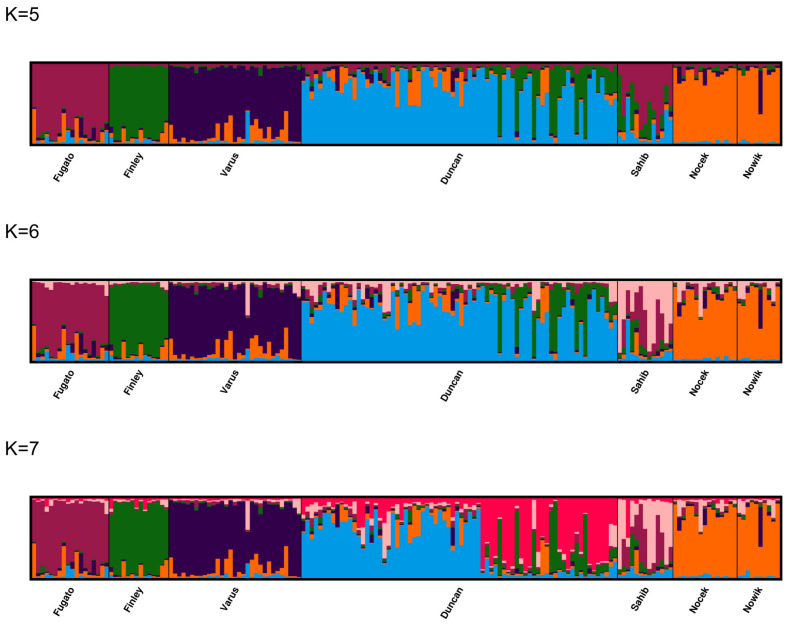
The major modes of CLUMPAK plots are shown for K = 5–7 from 20 independent STRUCTURE runs with K = 1–9 using 175 horses from seven paternal half-sib groups of Dülmen wild horses. Each individual is represented by a vertical line divided into K colors, where K is the number of clusters assumed and the colors show the consensus solutions for individual proportions of cluster memberships. Populations are separated by black lines. Paternal half-sib groups include male progeny of the sires Fugato 34 (Fugato), Finley 58 (Finley), Varus, Duncan, Sahib, Nocek, and Nowik.

The major modes from the post-processed population structure results are shown for K = 5–7 in Figure 6 and for K = 1–9 in Supplementary Materials Figure S3 based on CLUMPAK [40,41]. With K = 5–9, the progeny of Fugato 34, Finley 58, and Varus constitute their own clusters, whereas the offspring of Sahib mostly share two clusters with membership coefficients <0.5. The progeny of Duncan share mostly one cluster, but in a few iterations also two different clusters for K = 5 and 6. The progeny of Nocek and Nowik share either the same (particularly for K = 5–6 and 8) or the same two different clusters or even two different clusters (particularly for K = 7). Therefore, a substructure with five clusters appears as most likely, which is also supported by the MedMed K and MedMean K statistics at thresholds of 0.5 and 0.6. These five clusters are constituted by the offspring of Fugato 34, Finley 58, Varus, Duncan, and the common offspring of the two stallions Nocek and Nowik.

**Figure 7:**
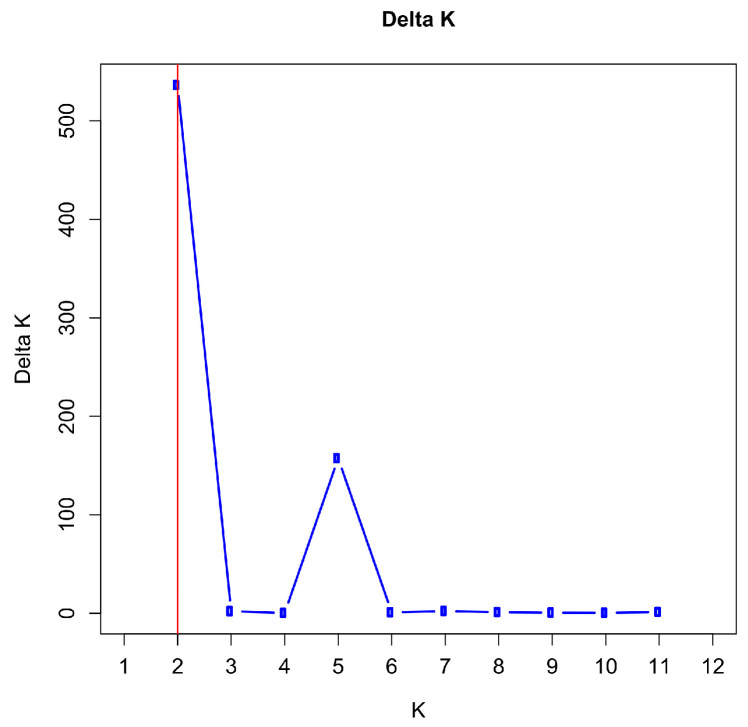
Plot of ΔK (Delta K) values from 20 independent STRUCTURE runs for K = 1–12 using 29 microsatellite genotypes of 10 subpopulations by paternal half-sib groups and birth years of Dülmen wild horses. The maximum value was achieved by K = 2, which is indicated by a red vertical line, and the next highest value was achieved at K = 5.

We further analyzed the substructure, when the birth cohorts of the offspring from the stallions Varus and Duncan were distinguished. The progeny of Varus were divided into two subpopulations (Varus-2011 and Varus-2014 for the birth years 2011 and 2014) and the progeny of Duncan into three subpopulations (Duncan-2011, Duncan-2012, and Duncan-2013 for the birth years 2011, 2012, and 2013). We performed 20 iterations for K = 1–12. The most likely number of clusters was two as determined by the maximum value for ΔK between K = 2 and 11 (Figure 7 and Supplementary Materials Table S14). With K = 2, ΔK reaches its maximum value with 532.22 and the next highest value is at K = 5 with 157.42. The mean of the likelihood function reaches its maximum with −21317.34 at K = 5 (Supplementary Materials Figure S4). For runs with other K values, ΔK reached much smaller values. 

**Figure 8:**
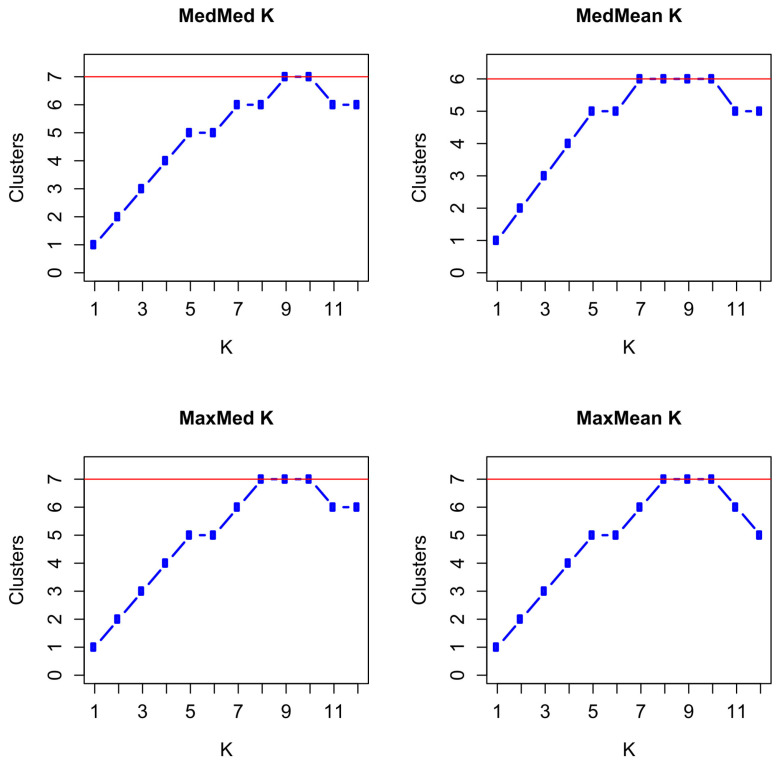
Plots of the medians (Med) and means (Mean) of the median (Med) and maximum (Max) number of inferred proportions of memberships to clusters with a threshold of 0.5. The red line indicates the maximum values reached by the MedMed K, MedMean K, MaxMed K, and MaxMean K statistics for the 10 subpopulations by paternal half-sib groups and birth years of Dülmen wild horses.

For the statistics MaxMed K and MaxMean K, we obtained maxima for K = 8–10 with seven clusters, and for the statistic MedMed K, we obtained maxima for K = 9–10 with seven clusters at a threshold of 0.5, while for the statistic MedMean K, we obtained maxima for K = 7–10 with six clusters at a threshold of 0.5 (Figure 8 and Supplementary Materials Table S15). Increasing the threshold to 0.6, resulted in MedMed K, MedMean K, and MaxMean K with K = 7 in six clusters, and in MaxMed K with K = 8 in seven clusters. With a threshold of 0.7, the maxima were obtained at K = 7 with five clusters for MedMed K, MedMean K, and MaxMean K, but with six clusters for MaxMed K.

**Figure 9:**
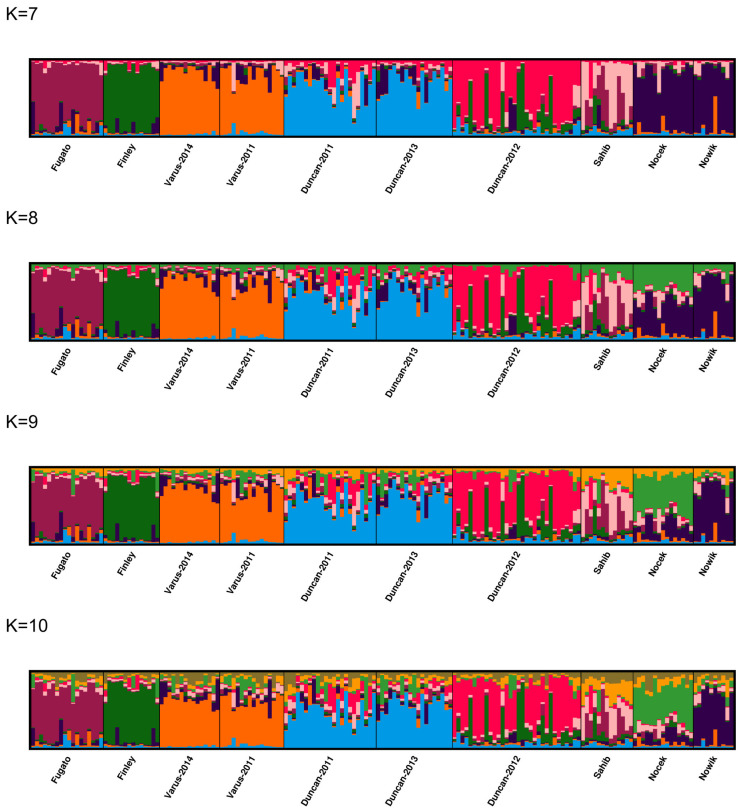
The figure shows the major modes of the CLUMPAK plots for K = 7–10 from 20 independent STRUCTURE runs from K = 1–12 using 175 horses from 10 groups of paternal half-sibs by birth cohorts of Dülmen wild horses. Each individual is represented by a vertical line divided into K colors, where K is the number of clusters assumed and the colors show the consensus solutions for individual proportions of cluster memberships. Subpopulations are separated by black lines. Paternal half-sib groups include male progeny of the sires Fugato 34 (Fugato), Finley 58 (Finley), Varus-2011, and Varus-2014 for the birth years 2011 and 2014, Duncan-2011, Duncan-2012, and Duncan-2013 for the birth years 2011, 2012, and 2013, Sahib, Nocek, and Nowik.

The major modes from the post-processed population structure results are shown for K = 7–10 in Figure 9 and for K = 1–12 in Supplementary Materials Figure S5 based on CLUMPAK [40,41]. With K = 7–10, the progeny of Fugato 34, Finley 58, and Varus from both birth years constitute their own clusters, whereas the offspring of Sahib share 2–4 clusters with membership coefficients of <0.4. A larger proportion of the progeny of Duncan from the birth years 2011 and 2013 shares one cluster, which is different to the cluster that is shared by a larger proportion of the progeny of Duncan from the birth years 2013 and 2012. In addition, a minor proportion from each of the 2011 and 2013 birth years, as well as from the 2013 and 2012 Duncan offspring, share another second cluster each. The progeny of Nocek and Nowik share either the same (particularly for K = 7–8) or the same two different clusters or even two different clusters (particularly for K = 9–10). Therefore, a substructure with 6–7 clusters appears as most likely, which is also supported by the MedMed K and MedMean K statistics at thresholds of 0.5 and 0.6. These 6–7 clusters are constituted by the offspring of Fugato 34, Finley 58, Varus (2011, 2014), Duncan (2011 and 2013), Duncan (2012), Nocek, and Nowik. A moderate part of the offspring of Finley 58 and Duncan (2012), which are sired in the same 2011 breeding season, share a cluster with membership coefficients of about 0.20, whereas a similar overlap of membership coefficients from the progeny of Varus (2011) and Duncan (2011) from the same breeding season in 2010 could not be found.

## 4. Discussion

The use of 2–3 stallions in free natural service per breeding season may have an impact on the future development of the genetic diversity of the Dülmen wild horse in the Merfelder Bruch near Dülmen. Our results using neighbor-joining dendrograms, genetic distances, and Bayesian population clustering indicated that stallions had an influence on the substructure and the further development of the genetic diversity in this herd of wild horses. The means of the posterior probabilities ln P(G|K) from 20 independent STRUCTURE runs as well as the MedMed K and MedMean K statistics support five different clusters with the stallions Fugato 34, Finley 58, Varus, and Duncan each in their own clusters and Nocek and Nowik in a joint cluster. The progeny of Sahib have a closer relationship with the progeny of Fugato 34, Finley 58, and Duncan, as Sahib is the grandsire of these stallions. Thus, the progeny of Sahib also jointly cluster with the clusters of his grandsons.

The ΔK method seems to underestimate the uppermost level of population structure, which may be due to the higher migration rates between the offspring groups, lower differentiation between the paternal half-sib groups and even unequal sampling per half-sib-group [35,36,37,38]. Migration rates are increasing with closer relationships among the stallions, and in the case of common male ancestors, migration starts from the most common ancestors to all of the different stallions and their offspring who are descending from the respective ancestor or ancestors. Therefore, particularly common ancestors that also sired foals in the Dülmen wild horse herd may contribute to the peak value of ΔK at K = 2 [35,36,37,38], resulting in an underestimation of the level of population structure. The pedigrees of the seven stallions used for STRUCTURE analyses revealed such a situation for stallions under analysis who are common ancestors of stallions with progeny in the present data set. The stallion Sahib had progeny and his sons Fugato 34, Finley 58, and Duncan also had progeny in the present data set.

The population structure, when distinguishing the birth years for the progeny of Varus and Duncan, revealed some interesting aspects on the mares’ influence (Supplementary Materials Table S16). The progeny of Duncan from the birth years 2011 and 2012 reach membership coefficients > 0.5 for different clusters, whereas the progeny of Duncan from the birth years 2013 share clusters with their paternal half sibs from the preceding two years with membership coefficients < 0.5. The offspring of Varus from the birth years 2011 and 2014 share the same cluster. A possible reason for this pattern may be attributed to the mating behavior of the Dülmen wild horses. When stallions are employed in consecutive breeding seasons, then they cannot sire the same mares, because most mares do not reproduce in consecutive years and leave out a consecutive pregnancy [1,42,43]. The example of Varus lets us suppose that this stallion may have had preference for the same mares and the same mares for this stallion after a break of two years. The females descending from the same maternal ancestor live together in family units of about 8–12 horses in this herd [1,42,43]. This maternal family structure could also influence the mating behavior of stallions in such a way that stallions may sire more than one mare of these family units [1,42,43]. Therefore, the selection of mating partners may not be completely random across all mares that can reproduce in a breeding season. Particularly, when there is competition among stallions which are employed in the same period, stallions are avoiding fights [1,42,43], and the reason for this may be due to the observation that stallions are arranged according to the maternal family units [1,42,43]. Therefore, the genetic distances and different cluster memberships of the offspring of the stallions may also be caused by differences of the maternal lines. The maternal lines cannot be analyzed because there is no way to access the female population of the Dülmen wild horse in the Merfelder Bruch [1,4].

In agreement with previous reports, we found significant inter-line differences using F_ST_, Ds, and D_US_ [10,16,17]. The F_ST_ values were similar on average (0.097) and smaller for the range between progeny groups (0.184) in the present study when compared with the Old Kladruber horse [16] and the Bilgoraj horse [17]. Overall multi-locus variability between paternal offspring was, in the present study, 9%, with the number of migrants per population (Nm) = 2.334, and multi-locus variability between paternal offspring in the Old Kladruber horse line was at 7%. The number of migrants varied in the present study between the progeny of the seven stallions with 10 and more progeny from 0.534 to 1.602, whereas in the Old Kladruber horse it ranged from 1.1 to 14.6 with F_ST_ values of 0.017 to 0.174 [16]. In contrast to the present study, a population structure was not found in Polish Konik due to the male founder lines [5].

We found high values for the number of alleles and observed, expected, and unbiased expected heterozygosity, and low values for Wright’s F_IS_ for all five birth cohorts of the Dülmen wild horses. Significant differences for measures of genetic diversity except for F_IS_ between two different birth year means could not be found. Also, a trend by birth years was not obvious. The means for the number of alleles, observed, expected, and unbiased expected heterozygosity, and Wright’s F_IS_ from our data were almost in the upper range as in previous reports for other horse breeds [3,4,5,6,7,8,9,10,11,12,13,14,15,16,17,18,19], even if there are some exceptions with horse breeds from the literature having higher values for genetic diversity [7,14,19]. Marker variability between birth cohorts was smaller than between progeny groups with a value of 4%, an overall F_ST_ value of 0.046, and an estimate of the number of migrants of 5.199. Differences between the birth cohorts are caused by the employment of different sires, the reproductive behavior of the mares, and the substructure of the herd in maternal units [1,42,43]. 

Strict breeding regulations in place since 1856 prevented the introgression of maternal genetics in the Dülmen wild horse population. Nine different Polish Konik stallions were siring Dülmen wild horse mares in the Merfelder Bruch herd from 1957 to 2004. The ancestor of five of the stallions in the present study was the Polish Konik stallion Kurs, born on 4 December 1960, registered under the breeding license number 5196. The stallion Kurs is the common ancestor of Darius, Duncan, Fugato 34, Finley 58, and Varus. The expected value for the proportion of genes from Kurs to his descendants reaches 0.781%. The common ancestor of Nocek, Nowik, and Nando was the Polish Konik stallion Tulipan, born 21 March 1979, with breeding license number 9 G Bł, from the Goraj sire line (PZHK Pedigree database, https://baza.pzhk.pl/en/horse/id/4914.html, accessed on 17 May 2024). The two full sibs are Polish Koniks and Nando is an F_1_ individual from a Dülmen wild horse mare and a Polish Konik stallion. The effects on the genetic make-up of these two common Polish Konik ancestors could be largely eliminated in the 2011–2014 birth cohorts by backcrossing with mares from the Dülmen wild horse herd. The genetic distance measures and Bayesian clustering thus clearly showed the influence of the Polish Konik stallions Nocek and Nowik, while an effect of the distant Polish Konik ancestor Kurs, which dates back seven generations, was not seen in the 2011–2014 birth cohorts. Since Polish Koniks cannot be differentiated by sire lines [5], it may be assumed that the individual differences of the Polish Konik ancestors in their genotypes should have only a rather small influence on the size of the genetic distance measures and cluster memberships. Maintaining the Dülmen wild horse population under natural harsh conditions without stabling or human interventions and with exclusively grassland pastures has contributed to the uniqueness of this wild horse herd and a careful selection of stallions that can withstand these conditions.

Continuous monitoring using dense SNP-arrays [44] and mtDNA markers [45,46] should be helpful to control breeding decisions and the fitness of stallions through the rate of male foals per day of employment in the breeding season. The expected inbreeding load of the future progeny through the potential candidates can be derived prior to their use in breeding [47] and can be an additional tool, alongside phenotypic criteria, such as physical development, temperament, and character, to keep genetic diversity as high as possible. The limitation of this study population arises from the fact that fillies and mares could not be genotyped to decipher their maternal ancestors and their effects on the genetic diversity.

## 5. Conclusions

In conclusion, our study demonstrated the significant effects of stallions on the Dülmen wild horse population. Therefore, a careful selection of stallions from the Dülmen wild horse herd is essential to maintain genetic diversity and fitness for the future generations. The number of the progeny of stallions is mainly determined by the duration of the period in which stallions are allowed to sire foals. Stallions significantly differ in their genotypes and therefore, only those young stallions should be selected that maintain the genetic diversity in their progeny on the same level as in the previous generations. Fluctuations in measures of genetic diversity between years may be possible due to non-random mating or other unknown factors. Stallions should be employed for not more than three consecutive breeding seasons to reduce the effect of single sires. Full and half sibs should not be employed in the same breeding season, as such related stallions seem to share a large proportion of the maternal units resulting in small genetic distances. A similar pattern was seen for stallions when siring in the third year or after a break of two years. In order to maintain genetic diversity, a nearly equal number of offspring per stallion should be aimed for. However, there might unforeseen reasons (stallion accidents and unexpected problems to sire mares) why this is not possible for each stallion. An extension of the study population is not possible due to the restrictions for sampling of fillies and mares.

## Figures and Tables

**Table 1:** Number of stallions, mares, foals born alive, and male foals at auctions.

Year of Siring	Birth Year	Year of Auction	Stallions	Mares	Foals Born Alive	Male Foals at Auctions
2001	2002	2003	3	238	107	38 (36%)
2010	2011	2012	3	~350	122	41 (34%)
2011	2012	2013	2	~350	133	46 (35%)
2012	2013	2014	3	406	65	27 (42%)
2013	2014	2015	2	370	92	33 (36%)
Total			9		519	185 (36%)

**Table 2:** Paternity of male foals from the birth years 2002, 2011, 2012, 2013, and 2014.

Stallion	2002	2011	2012	2013	2014	Total
Nocek	15	-	-	-	-	15
Nowik	10	-	-	-	-	10
Sahib	13	-	-	-	-	13
Varus	-	16	-	-	15	31
Duncan	-	23	32	19	-	74
Nando	-	2	-	-	-	2
Finley 58	-	-	14	-	-	14
Darius	-	-	-	5	-	5
Fugato 34	-	-	-	3	18	21
Total	38	41	46	27	33	185

**Table 3:** Sample size (N), observed (H_o_), expected (H_E_) heterozygosity, unbiased expected (uH_E_) heterozygosity, the mean number of alleles per locus (MNA), the mean number of effective alleles (N_e_), Shannons’s information index (I), and Wright’s F_IS_ (F_IS_) averaged over 29 microsatellites per each of the nine paternal male half-siblings from 185 Dülmen wild horses and across all paternal male half-sib groups from 185 male Dülmen wild horses sampled at auctions.

Population	N	H_o_	H_e_	uH_e_	MNA	N_e_	I	F_IS_
Sahib	13	0.692	0.608	0.635	4.655	2.908	1.171	−0.141
Darius	5	0.731	0.617	0.686	3.793	2.868	1.128	−0.186
Duncan	74	0.682	0.613	0.617	5.897	2.939	1.218	−0.118
Finley 58	14	0.678	0.593	0.616	4.103	2.797	1.098	−0.146
Fugato 34	21	0.666	0.601	0.616	5.069	2.766	1.157	−0.111
Varus	31	0.684	0.609	0.619	5.276	2.883	1.183	−0.122
Nocek	15	0.704	0.602	0.627	4.621	2.904	1.163	−0.162
Nowik	10	0.681	0.584	0.618	4.310	2.793	1.115	−0.169
Nando	2	0.672	0.474	0.644	2.310	2.083	0.736	−0.412
Total (Mean)	185	0.688	0.589	0.631	4.448	2.771	1.108	−0.173
Total (SE)	-	0.014	0.010	0.011	0.102	0.057	0.023	0.016

**Table 4:** Sample size (N), observed (H_o_) and expected (H_E_) heterozygosity, unbiased expected (uH_E_) heterozygosity, the mean number of alleles per locus (MNA), the mean number of effective alleles (N_e_), Shannons’s information index (I), and Wright’s F_IS_ (F_IS_) averaged over 29 microsatellites as well as estimated effective population size (N_eff_) and its 95% confidence intervals (N_eff_-CI) by the birth cohorts 2002 and 2011–2014 and across birth cohorts from 185 male Dülmen wild horses sampled at auctions.

Birth Cohort	N	H_o_	H_e_	uH_e_	MNA	N_e_	I	F_IS_	N_eff_	N_eff_-CI
2002	38	0.694	0.650	0.660	5.690	3.448	1.321	−0.074	67.2	53.7–88.0
2011	41	0.699	0.638	0.646	5.448	3.122	1.261	−0.087	65.5	54.1–81.8
2012	46	0.681	0.620	0.627	5.276	2.998	1.203	−0.102	95.7	74.8–129.9
2013	27	0.679	0.621	0.633	5.138	2.932	1.217	−0.095	60.9	45.2–90.1
2014	33	0.660	0.644	0.654	5.207	3.074	1.249	−0.024	44.8	37.1–55.6
Total (Mean)	185	0.683	0.635	0.644	5.352	3.115	1.250	−0.076	92.6	86.7–99.1
Total (SE)	-	0.014	0.012	0.012	0.120	0.087	0.028	0.011	-	-

## Data Availability

Restrictions apply to the availability of these data. Samples were obtained from Dülmen wild horses in the Merfelder Bruch with the allowance of Rudolph Herzog von Croÿ and are available from the authors upon reasonable request and with the permission of the horse owner.

## Supplementary Materials

The following supporting information can be downloaded at: https://www.mdpi.com/article/10.3390/ani14192904/s1, Figure S1. Individual-animal-based neighbor-joining dendrogram for the birth cohorts and the nine stallions used in the respective breeding seasons. Birth cohorts are represented in the same color and the stallions are marked by black color. Figure S2. Plots for the means of the posterior probabilities ln P(G|K) for K = 1–9. Figure S3. Major modes of CLUMPAK plots for K = 1–9 from 20 independent STRUCTURE runs with K = 1–9 using 175 horses from seven paternal half-sib groups of Dülmen wild horses. Each individual is represented by a vertical line divided into K colors, where K is the number of clusters assumed and the colors show the consensus solutions for individual proportions of cluster memberships. Populations are separated by black lines. Paternal half-sib groups include male progeny of the sires Fugato 34 (Fugato), Finley 58 (Finley), Varus, Duncan, Sahib, Nocek, and Nowik. Figure S4. Plots for the means of the posterior probabilities ln P(G|K) for K = 1–12. Figure S5. Major modes of the CLUMPAK plots for K = 1–12 from 20 independent STRUCTURE runs from K = 1–12 using 175 horses from 10 groups of paternal half sibs by birth cohorts of Dülmen wild horses. Each individual is represented by a vertical line divided into K colors, where K is the number of clusters assumed and the colors show the consensus solutions for individual proportions of cluster memberships. Subpopulations are separated by black lines. Paternal half-sib groups include male progeny of the sires Fugato 34 (Fugato), Finley 58 (Finley), Varus-2011, and Varus-2014 for the birth years 2011 and 2014, Duncan-2011, Duncan-2012, and Duncan-2013 for the birth years 2011, 2012, and 2013, Sahib, Nocek, and Nowik. Table S1. Characteristics of the 29 autosomal microsatellite markers analyzed in 185 Dülmen wild horses from the Merfelder Bruch. Number of alleles (N_A_), observed heterozygosity (H_o_), expected heterozygosity (H_E_), unbiased expected heterozygosity (H_E_), polymorphism information content (PIC), allelic diversity (AD), F-statistics with F_IS_, F_IT_, and F_ST_, and number of migrants (Nm) are given. Table S2. Duration of the use of stallions in the Dülmen wild horse population in the Merfelder Bruch and the observed (H_o_) and expected heterozygosity (H_e_) of the stallions from 29 autosomal microsatellite markers. Table S3. Comparisons of microsatellite characteristics between the nine progeny groups in Dülmen wild horses using p values of t-tests for group means. Table S4. Pairwise population F_ST_ values for all nine paternal progeny groups. Table S5. Pairwise population matrix of Nei genetic distance for all nine paternal progeny groups. Table S6. Pairwise population matrix of Nei unbiased genetic distance for all nine paternal progeny groups. Table S7. Pairwise AMOVA estimates between the seven paternal progeny groups with 10 and more individuals. Table S8. Comparisons of microsatellite characteristics between the five birth cohorts of Dülmen wild horses using *p* values of *t*-tests for group means. Table S9. Pairwise population F_ST_ values for the birth cohorts. Table S10. Pairwise population matrix of Nei genetic distance for the birth cohorts. Table S11. Pairwise population matrix of Nei unbiased genetic distance for the birth cohorts. Table S12. Results from the analysis of 20 independent runs of STRUCTURE [31,32,33,34] with STRUCTURESELECTOR [40] to obtain the mean of the posterior probability ln P(G|K) (Ln P(K)) with its standard deviation (Stdev Ln P(K)), Ln′(K), |Ln″(K)| and ΔK (Delta K) for K = 1–9. The maximum of the mean Ln P(K) is reached at K = 5 and for ΔK at K = 2 and this row is highlighted in yellow. Table S13. Results from the analysis of 20 independent runs of STRUCTURE [31,32,33,34] with STRUCTURESELECTOR [40] to obtain estimates for the MedMed K, MedMean K, MaxMed K, and MaxMean K statistics with a threshold of 0.5, 0.6, and 0.7 for membership coefficients per cluster for the seven subpopulations of paternal half-sib groups from Dülmen wild horses. Table S14. Results from the analysis of 20 independent runs of STRUCTURE [31,32,33,34] with STRUCTURESELECTOR [40] to obtain the mean of the posterior probability ln P(G|K) (Ln P(K)) with its standard deviation (Stdev Ln P(K)), Ln′(K), |Ln″(K)| and ΔK (Delta K) for K = 1–12 of the 10 subpopulations of paternal half-sib groups by birth years from Dülmen wild horses. The maximum of the mean Ln P(K) is reached at K = 5 and for ΔK at K = 2 and this row is highlighted in yellow. Table S15. Results from the analysis of 20 independent runs of STRUCTURE [31,32,33,34] with STRUCTURESELECTOR [40] to obtain estimates for the MedMed K, MedMean K, MaxMed K, and MaxMean K statistics with a threshold of 0.5, 0.6, and 0.7 for membership coefficients per cluster for the 10 subpopulations of paternal half-sib groups by birth years from Dülmen wild horses. Table S16. Pairwise population distance measures (F_ST_, Nei genetic distances, Nei unbiased genetic distances) for seven paternal progeny groups, distinguished by birth years of their offspring.
