# Supplementary material for: Influence of Sires on Population Substructure in Dülmen Wild Horses
Source: Animals (Basel). 2024 Oct 9;14(19):2904. doi: 10.3390/ani14192904 (PMC11475081; doi:10.3390/ani14192904)
Supplement: Supplementary file 1 [file animals-14-02904-s001.zip › Supplementary-Materials-Figures-S1-S5.pdf]

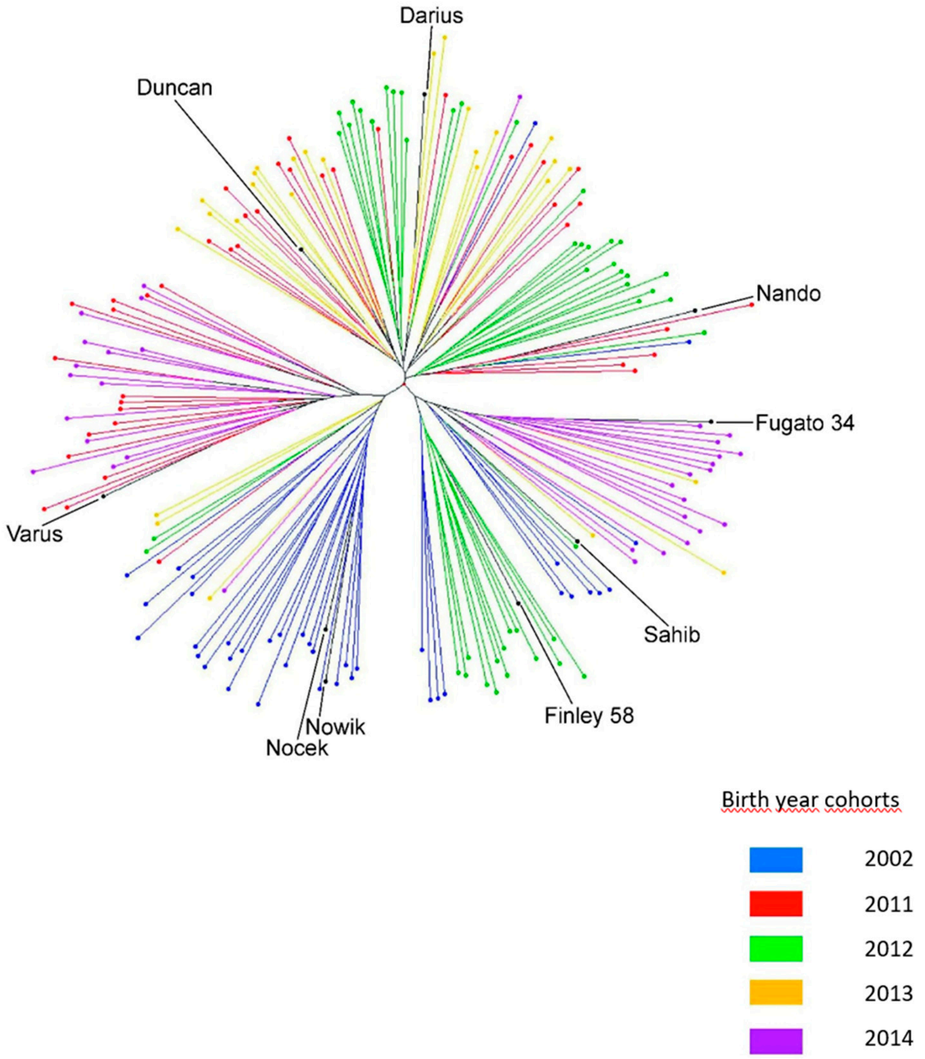

Figure S1. Individual-animal-based neighbour-joining dendogram for the birth cohorts and the 9 stallions used in the respective breeding seasons. Birth cohorts are represented in the same colour and the stallions are marked by black colour.

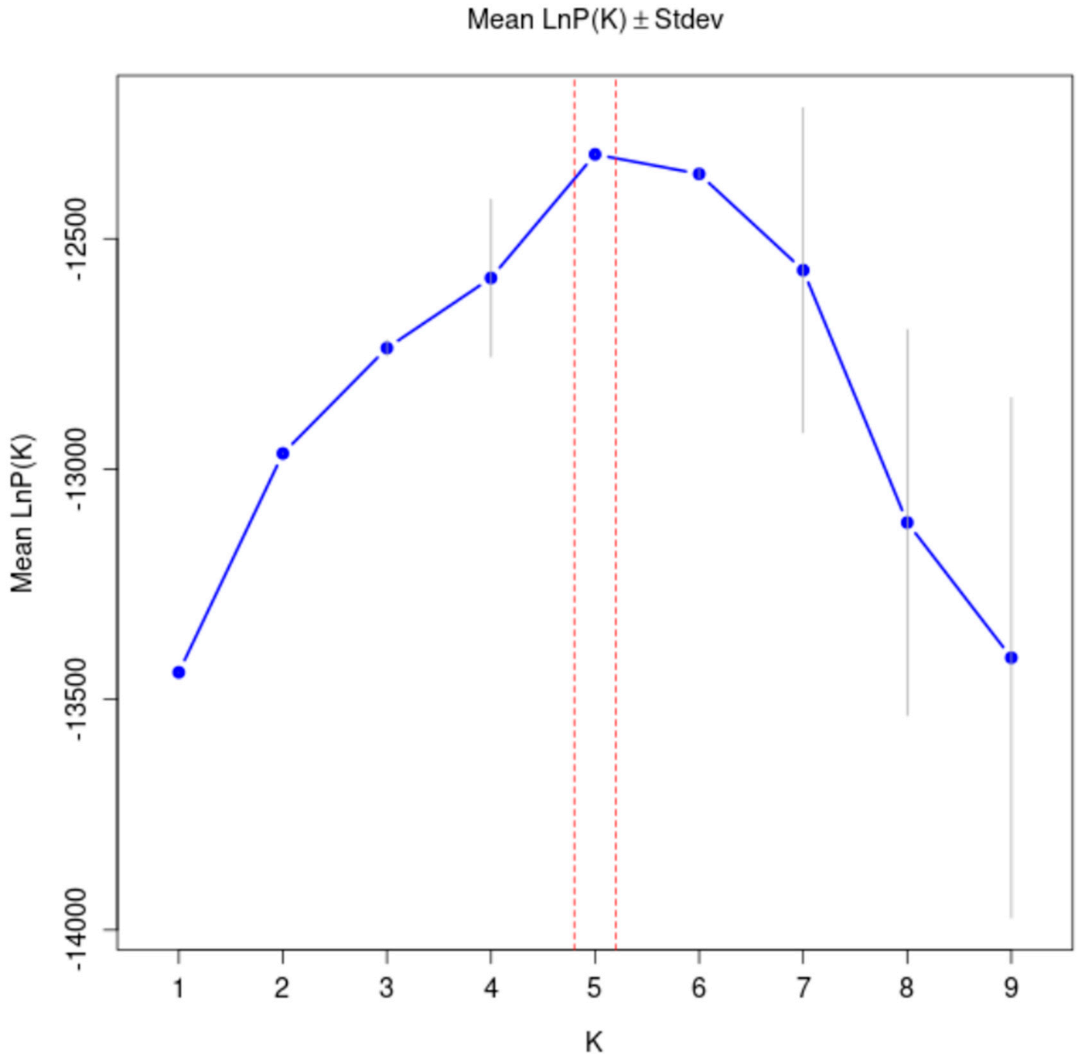

Figure S2. Plots for the means of the posterior probabilities ln P(G|K) for K = 1-9.

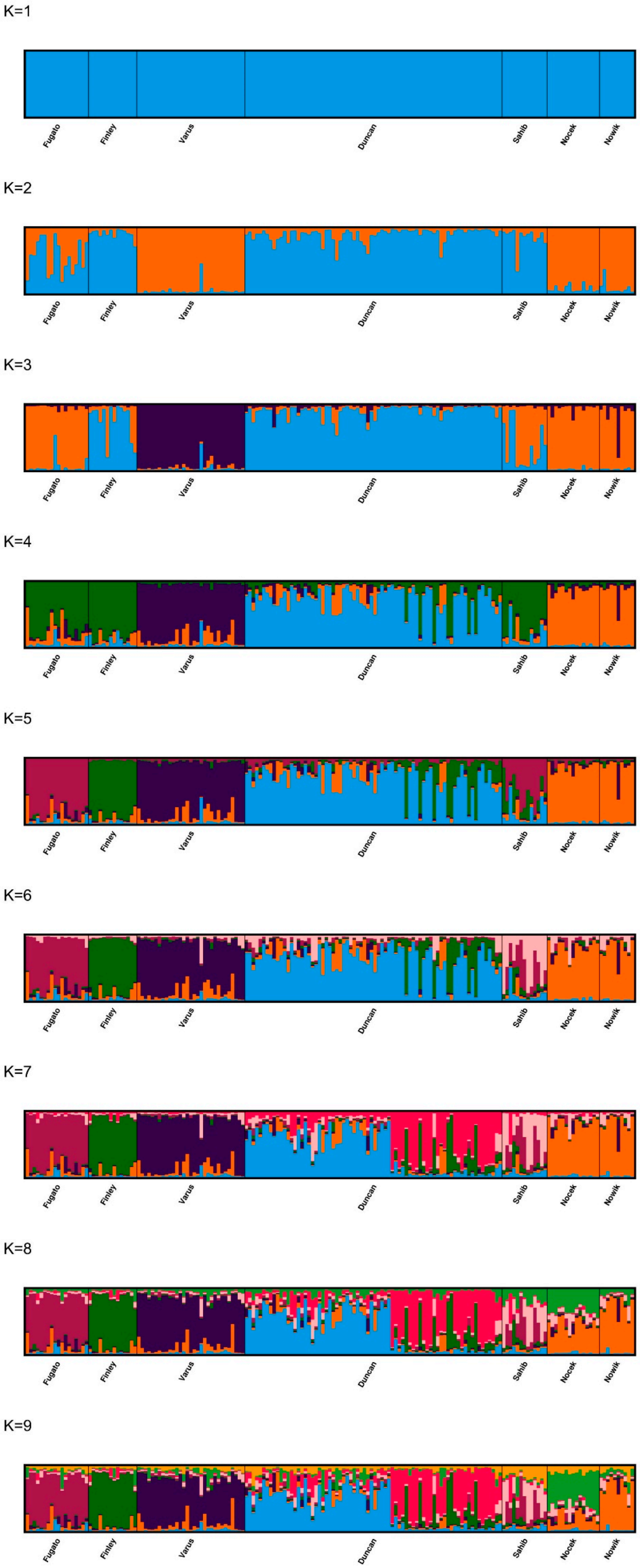
K=1
Fugato Finley Varus Duncan Sahib Nocek Nowik
K=2
Fugato Finley Varus Duncan Sahib Nocek Nowik
K=3
Fugato Finley Varus Duncan Sahib Nocek Nowik
K=4
Fugato Finley Varus Duncan Sahib Nocek Nowik
K=5
Fugato Finley Varus Duncan Sahib Nocek Nowik
K=6
Fugato Finley Varus Duncan Sahib Nocek Nowik
K=7
Fugato Finley Varus Duncan Sahib Nocek Nowik
K=8
Fugato Finley Varus Duncan Sahib Nocek Nowik
K=9
Fugato Finley Varus Duncan Sahib Nocek Nowik

Figure S3. Major modes of CLUMPAK plots for K = 1–9 from 20 independent STRUCTURE runs with K = 1–9 using 175 horses from 7 paternal half-sib groups of Dülmen wild horses. Each individual is represented by a vertical line divided into K colors, where K is the number of clusters assumed and the colors show the consensus solutions for individual proportions of cluster memberships. Populations are separated by black lines. Paternal half-sib groups include male progeny of the sires Fugato 34 (Fugato), Finley 58 (Finley), Varus, Duncan, Sahib, Nocek and Nowik.

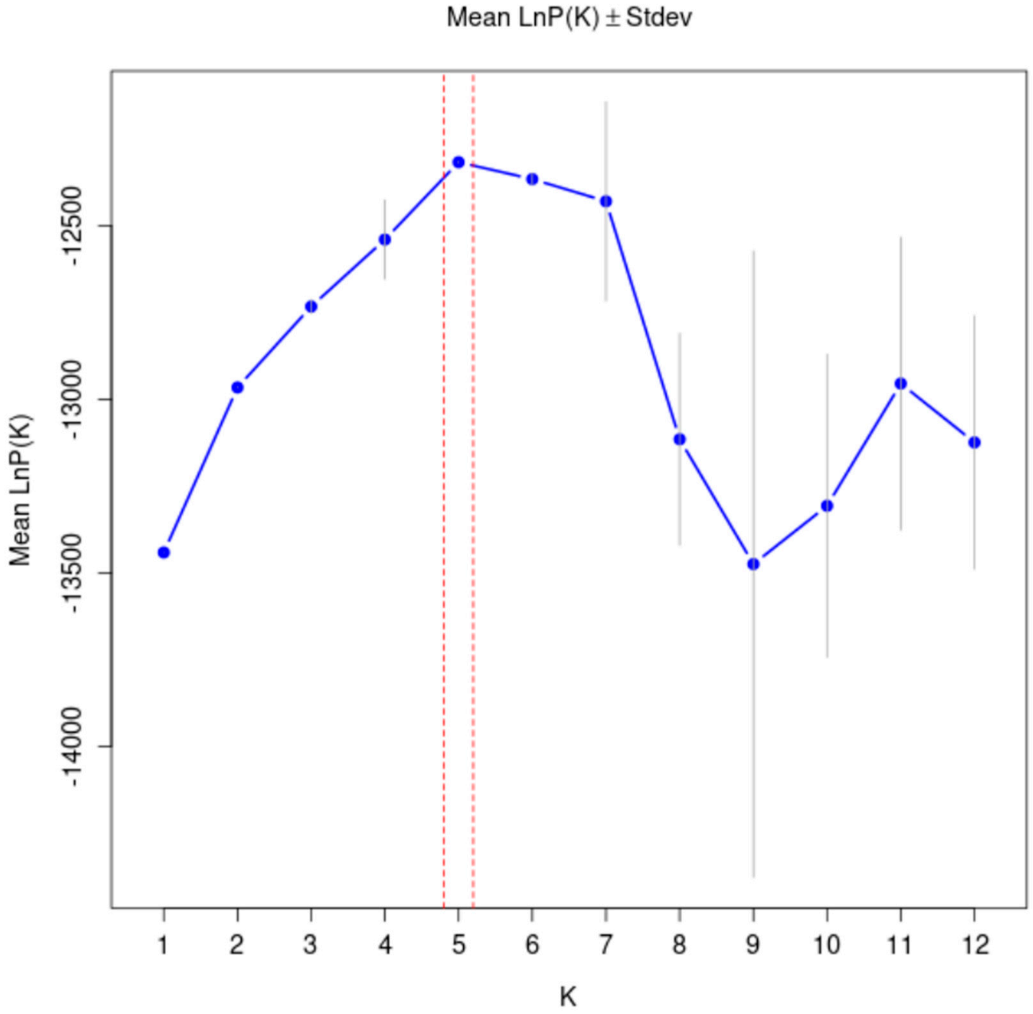

Figure S4. Plots for the means of the posterior probabilities ln P(G|K) for K = 1-12.

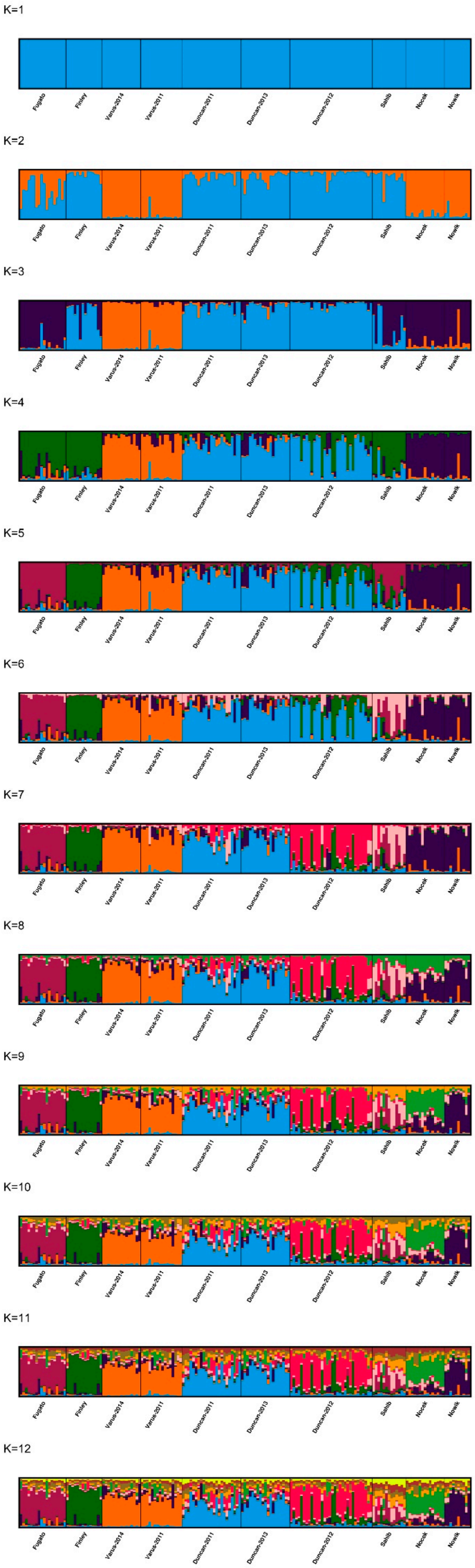
K=1
K=2
K=3
K=4
K=5
K=6
K=7
K=8
K=9
K=10
K=11
K=12
Fugato
Finley
Varus-2014
Varus-2011
Duncan-2011
Duncan-2013
Duncan-2012
Salib
Nocek
Nowik

Figure S5. Major modes of the CLUMPAK plots for K = 1–12 from 20 independent STRUCTURE runs from K = 1–12 using 175 horses from 10 groups of paternal half-sibs by birth cohorts of Dülmen wild horses. Each individual is represented by a vertical line divided into K colors, where K is the number of clusters assumed and the colors show the consensus solutions for individual proportions of cluster memberships. Subpopulations are separated by black lines. Paternal half-sib groups include male progeny of the sires Fugato 34 (Fugato), Finley 58 (Finley), Varus-2011 and Varus-2014 for the birth years 2011 and 2014, Duncan-2011, Duncan-2012 and Duncan-2013 for the birth years 2011, 2012 and 2013, Sahib, Nocek and Nowik.
