# Supplementary material for: Influence of Sires on Population Substructure in Dülmen Wild Horses
Source: Animals (Basel). 2024 Oct 9;14(19):2904. doi: 10.3390/ani14192904 (PMC11475081; doi:10.3390/ani14192904)
Supplement: Supplementary file 1 [file animals-14-02904-s001.zip › Supplementary-Materials-Tables-S1-S2.pdf]

Table S1. Characteristics of the 29 autosomal microsatellite markers analyzed in 185 Dülmen wild horses from the Merfelder Bruch. Number of alleles (N_A), observed heterozygosity (H_O), expected heterozygosity (H_E), unbiased expected heterozygosity (H_E), polymorphism information content (PIC), allelic diversity (AD), F-statistics with $F_{IS,}$ F_{IT} and F_{ST} and number of migrants (Nm) are given.

**Locus**	**N_A**	**H_O**	**H_E**	**uH_E**	**PIC**	**AR**	**Fis**	**Fit**	**Fst**	**Nm**
**AHT34**	6	0.6703	0.626	0.688	0.6469	0.6863	-0.102	0.030	0.120	1.833
**ASB17**	8	0.6919	0.682	0.816	0.7876	0.8142	-0.033	0.137	0.165	1.261
**COR007**	7	0.8222	0.658	0.757	0.7161	0.7554	-0.225	-0.062	0.133	1.630
**COR017**	6	0.7845	0.650	0.712	0.6553	0.7100	-0.286	-0.181	0.081	2.826
**COR018**	7	0.7622	0.667	0.731	0.7007	0.7288	-0.151	-0.030	0.105	2.125
**COR022**	4	0.6630	0.578	0.590	0.5246	0.5882	-0.166	-0.041	0.108	2.075
**COR024**	6	0.7213	0.554	0.704	0.6466	0.7022	-0.293	-0.006	0.222	0.876
**COR045**	8	0.6704	0.617	0.632	0.6054	0.6301	-0.097	0.031	0.117	1.886
**COR056**	11	0.7663	0.729	0.837	0.8154	0.8350	-0.107	0.026	0.119	1.845
**COR058**	8	0.8095	0.684	0.771	0.7416	0.7689	-0.280	-0.073	0.161	1.302
**COR069**	6	0.7633	0.623	0.720	0.6692	0.7175	-0.221	-0.087	0.110	2.032
**COR070**	9	0.7931	0.707	0.759	0.7199	0.7569	-0.197	-0.101	0.081	2.848
**COR071**	6	0.6739	0.552	0.631	0.5589	0.6297	-0.154	-0.021	0.115	1.922

**COR082**	6	0.7778	0.683	0.770	0.7320	0.7680	-0.133	0.013	0.129	1.689
**HMS07**	6	0.6023	0.614	0.662	0.6078	0.6600	-0.034	0.094	0.123	1.779
**HTG03**	5	0.6557	0.571	0.651	0.6065	0.6490	-0.058	0.067	0.117	1.879
**HTG06**	5	0.2595	0.242	0.237	0.2257	0.2359	-0.196	-0.121	0.062	3.773
**LEX07**	5	0.5163	0.421	0.455	0.4249	0.4539	-0.309	-0.150	0.122	1.802
**LEX33**	11	0.8023	0.700	0.832	0.8081	0.8298	-0.094	0.052	0.133	1.630
**LEX34**	4	0.5889	0.548	0.564	0.4825	0.5622	-0.149	-0.038	0.097	2.326
**LEX63**	6	0.5848	0.554	0.606	0.5248	0.6040	-0.063	0.065	0.120	1.825
**LEX68**	7	0.7253	0.596	0.733	0.6990	0.7312	-0.115	0.029	0.129	1.692
**SGCV16**	5	0.4525	0.313	0.474	0.4335	0.4727	-0.108	0.053	0.145	1.472
**SGCV28**	4	0.7600	0.527	0.662	0.6048	0.6597	-0.316	-0.162	0.117	1.882
**TKY019**	6	0.7602	0.650	0.732	0.6825	0.7300	-0.243	-0.077	0.134	1.612
**CA425 (UCDEQ425)**	6	0.3146	0.363	0.324	0.3057	0.3232	-0.120	0.039	0.142	1.508
**UM011**	9	0.7650	0.709	0.754	0.7249	0.7522	-0.128	-0.018	0.097	2.315
**VHL20**	9	0.8865	0.674	0.814	0.7846	0.8117	-0.312	-0.119	0.147	1.450
**VHL209**	6	0.7747	0.590	0.751	0.7095	0.7491	-0.206	0.020	0.187	1.085

**Total**	6.621±0.345	0.683± 0.027	0.666± 0.027	0.668± 0.027	0.626± 0.027	0.666± 0.027	-0.169± 0.016	-0.022± 0.015	0.126± 0.006	1.868± 0.106

Table S2. Duration of the use of stallions in the Dülmen wild horse population in the Merfelder Bruch and the observed (H_O) and expected heterozygosity (H_e) of the stallions from 29 autosomal microsatellite markers.

Stallion	Year of covering	Covering period	H_O	H_e
Nocek	2001	17.04.-25.05.	0.690	0.345
Nowik	2001	17.04.-25.05.	0.621	0.310
Sahib	2001	28.05.-11.06.	0.724	0.362
Nando	2010	14.04.-18.04.	0.621	0.310
Duncan	2010	14.04.-21.05.	0.448	0.224
	2011	11.04.-20.05.		
	2012	23.04.-14.05.		
Varus	2010	19.04.-21.05.	0.690	0.345
	2013	15.04.-16.05.		
Finley 58	2011	11.04.-20.05.	0.621	0.310
Darius	2012	23.04.-04.05.	0.862	0.431
Fugato 34	2012	04.05.-14.05.	0.621	0.310
	2013	15.04.-16.05.		
