# Supplementary material for: Influence of Sires on Population Substructure in Dülmen Wild Horses
Source: Animals (Basel). 2024 Oct 9;14(19):2904. doi: 10.3390/ani14192904 (PMC11475081; doi:10.3390/ani14192904)
Supplement: Supplementary file 1 [file animals-14-02904-s001.zip › Supplementary-Materials-Tables-S12-S15.pdf]

Table S12. Results from the analysis of 20 independent runs of STRUCTURE [31-34] with STRUCTURESELECTOR [40] to obtain the mean of the posterior probability ln P(G|K) (Ln P(K)) with its standard deviation (Stdev Ln P(K)), Ln′(K), |Ln″(K)| and ΔK (Delta K) for K = 1–9 for the 7 subpopulations of paternal half-sib groups from Dülmen wild horses. The maximum for the mean Ln P(K) is reached at K = 5 and for ΔK at K = 2 and this row is highlighted in yellow.

K	Reps	Mean LnP(K)	Stdev LnP(K)	Ln'(K)	|Ln''(K)|	Delta K
1	20	-13441.05000	0.33325	NA	NA	NA
2	20	-12966.04000	0.58974	475.01000	246.25000	417.55917
3	20	-12737.28000	17.61207	228.76000	76.67500	4.35355
4	20	-12585.19500	170.40498	152.08500	116.16500	0.68170
5	20	-12316.94500	1.13577	268.25000	310.73000	273.58530
6	20	-12359.42500	10.18869	-42.48000	166.45500	16.33724
7	20	-12568.36000	352.16890	-208.93500	338.67000	0.96167
8	20	-13115.96500	418.14653	-547.60500	254.28000	0.60811
9	20	-13409.29000	563.93466	-293.32500	NA	NA

Table S13. Results from the analysis of 20 independent runs of STRUCTURE [31-34] with STRUCTURESELECTOR [40] to obtain estimates for the MedMed K, MedMean K, MaxMed K and MaxMean K statistics with a threshold of 0.5, 0.6 and 0.7 for membership coefficients per cluster for the 7 subpopulations of paternal half-sib groups from Dülmen wild horses.

Threshold=0.5

K	MedMed	MedMean	MaxMed	MaxMean	Reps
1	1	1	1	1	20
2	2	2	2	2	20
3	3	3	3	3	20
4	4	4	4	4	20
5	5	5	5	5	20
6	5	5	5	5	20
7	4	4	6	6	20
8	4	4	6	6	20
9	5	5	5	5	20
	MedMedK	MedMeaK	MaxMedK	MaxMeaK	
	5	5	6	6	

Threshold=0.6

K	MedMed	MedMean	MaxMed	MaxMean	Reps
1	1	1	1	1	20
2	2	2	2	2	20
3	3	3	3	3	20
4	4	4	4	4	20
5	5	5	5	5	20
6	5	5	5	5	20
7	4	4	6	5	20
8	4	4	4	4	20
9	4	4	4	4	20
	MedMedK	MedMeaK	MaxMedK	MaxMeaK	
	5	5	6	5	

Threshold=0.7

K	MedMed	MedMean	MaxMed	MaxMean	Reps
1	1	1	1	1	20
2	2	2	2	2	20
3	3	3	3	3	20
4	4	4	4	4	20
5	5	4	5	4	20
6	4	4	4	4	20
7	4	4	4	4	20
8	4	4	4	4	20
9	2	1	2	2	20
	MedMedK	MedMeaK	MaxMedK	MaxMeaK	
	5	4	5	4	

Table S14. Results from the analysis of 20 independent runs of STRUCTURE [31-34] with STRUCTURESELECTOR [40] to obtain the mean of the posterior probability ln P(G|K) (Ln P(K)) with its standard deviation (Stdev Ln P(K)), Ln′(K), |Ln″(K)| and ΔK (Delta K) for K = 1–12 of the 10 subpopulations of paternal half-sib groups by birth years from Dülmen wild horses. The maximum of the mean Ln P(K) is reached at K = 5 and for ΔK at K = 2 and this row is highlighted in yellow.

K	Reps	Mean LnP(K)	Stdev LnP(K)	Ln'(K)	|Ln''(K)|	Delta K
1	20	-13441.13000	0.18093	NA	NA	NA
2	20	-12966.04500	0.45128	475.08500	241.99000	536.22467
3	20	-12732.95000	19.85570	233.09500	40.06500	2.01781
4	20	-12539.92000	113.38803	193.03000	29.55000	0.26061
5	20	-12317.34000	1.72303	222.58000	271.24000	157.42010
6	20	-12366.00000	17.75352	-48.66000	15.27500	0.86039
7	20	-12429.93500	286.06155	-63.93500	621.31000	2.17195
8	20	-13115.18000	304.92731	-685.24500	325.88000	1.06871
9	20	-13474.54500	900.94023	-359.36500	527.14500	0.58511
10	20	-13306.76500	435.59071	167.78000	184.18500	0.42284
11	20	-12954.80000	421.32526	351.96500	521.42500	1.23758
12	20	-13124.26000	364.27367	-169.46000	NA	NA

Table S15. Results from the analysis of 20 independent runs of STRUCTURE [31-34] with STRUCTURESELECTOR [40] to obtain estimates for the MedMed K, MedMean K, MaxMed K and MaxMean K statistics with a threshold of 0.5, 0.6 and 0.7 for membership coefficients per cluster for the 10 subpopulations of paternal half-sib groups by birth years from Dülmen wild horses.

Threshold=0.5

K	MedMed	MedMean	MaxMed	MaxMean	Reps
1	1	1	1	1	20
2	2	2	2	2	20
3	3	3	3	3	20
4	4	4	4	4	20
5	5	5	5	5	20
6	5	5	5	5	20
7	6	6	6	6	20
8	6	6	7	7	20
9	7	6	7	7	20
10	7	6	7	7	20
11	6	5	6	6	20
12	6	5	6	5	20
	MedMedK	MedMeaK	MaxMedK	MaxMeaK	
	7	6	7	7	

Threshold=0.6

K	MedMed	MedMean	MaxMed	MaxMean	Reps
1	1	1	1	1	20
2	2	2	2	2	20
3	3	3	3	3	20
4	4	4	4	4	20
5	5	5	5	5	20
6	5	5	5	5	20
7	6	6	6	6	20
8	6	5	7	5	20
9	6	5	6	5	20
10	5	4	5	4	20
11	2	1	5	3	20
12	1	1	3	3	20
	MedMedK	MedMeaK	MaxMedK	MaxMeaK	
	6	6	7	6	

Threshold=0.7

K	MedMed	MedMean	MaxMed	MaxMean	Reps
1	1	1	1	1	20
2	2	2	2	2	20
3	3	3	3	3	20
4	4	4	4	4	20
5	5	5	5	5	20
6	5	4	5	4	20
7	5	5	6	5	20
8	4	4	4	4	20
9	2	2	3	2	20
10	1	1	1	1	20
11	1	0	1	1	20
12	1	0	1	0	20
	MedMedK	MedMeaK	MaxMedK	MaxMeaK	
	5	5	6	5	
